# Bone quality changes as measured by Raman and FTIR spectroscopy in primiparous cows with humeral fracture from New Zealand

**DOI:** 10.3389/fvets.2023.1063427

**Published:** 2023-02-09

**Authors:** Alvaro Wehrle-Martinez, Mark R. Waterland, Rafea Naffa, Kevin Lawrence, Penny J. Back, Chris W. Rogers, Keren Dittmer

**Affiliations:** ^1^School of Veterinary Sciences, Massey University, Palmerston North, New Zealand; ^2^School of Natural Sciences, Massey University, Palmerston North, New Zealand; ^3^MacDiarmid Institute for Advanced Materials and Nanotechnology, Victoria University, Wellington, New Zealand; ^4^Fonterra Research and Development Centre, Palmerston North, New Zealand; ^5^School of Agriculture and Environmental Sciences, Massey University, Palmerston North, New Zealand

**Keywords:** cows, humeral fracture, Raman, FTIR spectroscopy, band ratios, osteoporosis

## Abstract

The occurrence of spontaneous humeral fractures in primiparous dairy cows from New Zealand prompted the study of bone material from affected cows to further characterize this condition and to outline a likely pathogenesis. Previous studies indicate that these cows developed osteoporosis due to periods of suboptimal bone formation followed by increased bone resorption during the period of lactation complicated by copper deficiency. We hypothesized that there are significant differences in the chemical composition/bone quality in bones from cows with spontaneous humeral fracture compared to cows without humeral fractures. In this study, Raman and Fourier transform infrared spectroscopy band ratios were, for the first time, measured, calculated, and compared in bone samples from 67 primiparous dairy cows that suffered a spontaneous fracture of the humerus and 14 age-matched post-calving cows without humeral fractures. Affected bone showed a significantly reduced mineral/matrix ratio, increased bone remodeling, newer bone tissue with lower mineralization and, lower carbonate substitution, and reduced crystallinity. As such, is likely that these have detrimentally impacted bone quality and strength in affected cows.

## 1. Introduction

An increased incidence of spontaneous humeral fractures in primiparous dairy cows in New Zealand has prompted investigations characterizing different parameters that influence bone tissue strength in bone samples from normal and fractured bone material using a variety of techniques (1–3). For example, histological analysis of bone samples from primiparous cows with humeral fracture determined that cows with humeral fractures developed osteoporosis characterized by decreased bone volume, abnormal trabecular architecture, presence of growth arrest lines, thinner cortex with increased resorption in the cortex and distal humerus (1). These findings were associated with periods of poor bone formation (probably due to protein-calorie undernutrition), increased bone resorption associated with lactation, and periods of copper deficiency (1, 4). Peripheral quantitative computed tomography of the mid-diaphysis of the humerus showed that fractured animals had reduced cortical bone mineral density which reduced the stress-strain index (3).

Bone strength is determined by multiple factors including bone architecture, bone tissue quality, and bone mass/quantity (5, 6). For example, the amount of the bone organic matrix and mineral crystal hydroxyapatite [Ca_10_(PO_4_)_6_(OH)_2_] influences bone physicochemical properties (7, 8). Similarly, differences in the rate and site of bone turnover (the process of bone resorption followed by new bone formation) can affect bone quality and quantity (5, 7). Furthermore, the ability of bone to resist fractures (or bone toughness) is dependent on the quality and quantity of the matrix and mineral component of bone (6).

Because variations in the chemical composition of bone can influence the structural quality of bone, assessment of bone chemical composition (matrix and mineral components) using Raman and Fourier transform infrared spectroscopy (FTIR) can provide valuable and novel information for the characterization of humeral fractures in dairy cows in New Zealand (5, 6, 9–11). These techniques rely on the unique vibrational characteristic of each chemical component that is forming bone and are reliable methods to evaluate the relative chemical composition of bone (6, 9).

We hypothesized that there are significant differences in the chemical composition and bone quality in bones from cows with spontaneous humeral fracture compared to cows without humeral fractures. To test this, this study evaluated band intensity ratios measured by using Raman spectroscopy and attenuated total reflectance (ATR)-FTIR spectroscopy from humeral cortical and trabecular bone samples from primiparous cows with humeral fractures and age-matched cows without humeral fractures.

## 2. Materials and methods

### 2.1. Study design, sample collection, and preparation of bone samples

This was a case-control study using a convenience sample of 67 fractured (affected group) and 14 non-fractured (control group) humeri from a total of 81 primiparous dairy cows. The case definition for enrolling an animal in the affected group was a dairy cow of any breed, at least 2 years old, which had suffered a spontaneous fracture of the humerus, without any history of trauma, within 6 months of calving. The humeral bone samples from the affected animals were provided by farmers and veterinarians who after reporting a bovine fracture which met the case definition were asked to collect a bone sample post-mortem, as such animal ethical approval was not required. Control samples were obtained from an animal rendering plant (Wallace Corporation, New Zealand) and Massey University School of Veterinary Science postmortem service (Palmerston North, New Zealand). Samples were taken from dairy cows of any breed, with an ear tag indicating they were at least 2 years old, who had calved recently (udder consistent with lactating) and had been culled for reasons unrelated to bone fracture of the humerus or any other bone. From each control animal, a sample of the humerus was collected post-mortem. For both groups (affected and control), little information regarding sample handling and/or time between collection and reception was available and/or recorded in this study. Most samples from affected cows were sent overnight by courier.

**Figure 1:**
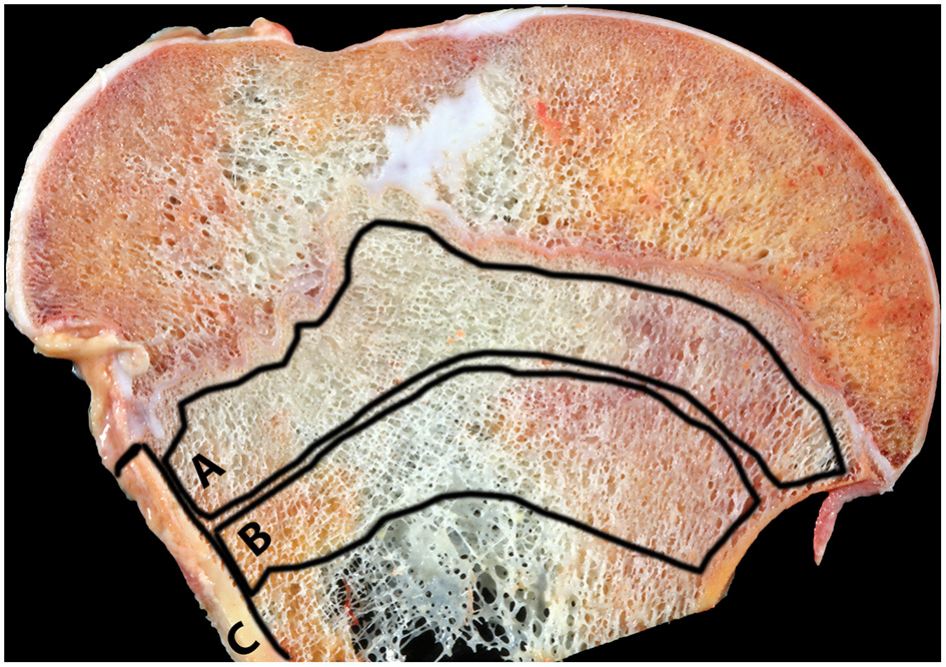
Humeral bone slab, affected cow, proximal humerus. The three locations selected for spectroscopic analysis are shown. A, primary spongiosa; B, secondary spongiosa; C, cortex.

From each humerus (*n* = 81), several bone slabs (~3–5 mm thick) from the proximal epiphysis and metaphysis were obtained using an industrial-grade band saw. One slab from the humerus was selected and then cleaned with high-pressure cold water to remove the bone marrow. From each slab, three locations were selected for analysis: the cortex, the primary and secondary spongiosa as shown in Figure 1. Each location was ground separately for 4 min using a cryogenic grinder filled with liquid nitrogen (6875 Freezer/Mill^®^, SPEX^®^ SamplePrep, Metuchen, NJ, USA). The grind protocol included two cycles each with a pre-cool step, a run step of 2 min, a cool step, and a second run time of 2 min. The impactor rate was set at 5 cycles per second. Once the cycle was finalized, powdered material was stored in Eppendorf tubes covered with aluminum foil at −80°C until further processing.

### 2.2. Raman spectra acquisition

For each cow bone sample (*n* = 81) and each location (cortex, primary and secondary spongiosa), three smaller (triplicates) portions of the powdered bone were mounted onto a glass slide and covered with a cover slip (22 × 22 mm) to form three thin films of randomly oriented powdered specimen. Thus, giving nine samples per cow.

Raman spectra were collected using a custom-made Raman microscope system with 532 nm monochromatic laser (Laser Quantum Torus 532). An Olympus IX-70 inverted fluorescence microscope body fitted a custom-manufactured 532 nm band-edge filter with a 12° incident angle (Iridian Technologies, Ottawa, Ontario, Canada) directed the laser into a 10× magnification objective (NA 0.25, Edmund Optics, Singapore) which provided a laser spot size of approximately 5 μm in diameter. Raman scattering was collimated by the same objective. Rayleigh scattering was partially rejected by the incident band-edge filter and a second 532 nm Raman edge-filter (150 cm^−1^ cut-off) placed immediately before the spectrograph provided complete Rayleigh rejection. The collimated Raman scattering was focused onto the entrance slit (50 μm) of a FERGIE spectrograph from Princeton/Teledyne Instruments equipped with a 1,024 × 256 pixel, thermoelectric cooling at −45°C charged coupled device (CCD) controlled Lightfield software (version 6.0.4.1611, Princeton Instruments, Trenton, NJ, USA).

Raman scattered light was collected over a spectral range of 200 to 4,000 cm^−1^, the resolution was 7–10 μm, with laser power set to 5–10 mW. One hundred and twenty individual frames were acquired for each replicate spectrum with an exposure time set to 1 second per frame. Acquisition of individual frames allowed for a quick and straightforward assessment of sample damage (i.e., compare the first and last frame for each replicate). No sample damage was observed using this method.

### 2.3. FTIR spectra acquisition

From each cow bone sample (*n* = 81) and for each location (cortex, primary, and secondary spongiosa), a small portion of powdered bone was placed onto the diamond crystal until fully covered. Background spectra were collected before each experiment, with no sample present on the diamond crystal. FTIR spectra of the powdered bone were collected using an FTIR spectrometer (Nicolet™ iS5™ FTIR Spectrometer, Thermo Scientific, MA, USA) with an iD7 diamond ATR attachment. OMNIC series software (v9.0) was used for data collection. Spectra were acquired in reflectance mode with a frequency region from 4000–400 cm^−1^ with 32 scans per spectrum and a spectral resolution of 2 cm^−1^. Spectra were sampled to give 14,936 data points for spectrum. No online corrections were applied to the collected data.

### 2.4. Spectral data processing

Spectral data (Raman and FTIR) was analyzed by bone location (cortex, primary and secondary spongiosa) using Python™ 3.9.× code (www.python.org) written in Jupyter notebooks (https://jupyter.org), or as scripts from the command line. Prior to peak fitting, individual spectra were normalized and a baseline correction (using a modified asymmetric least squares baseline algorithm) was applied (12). Background removal was achieved with an asymmetric least square algorithm modified for improved performance with broad overlapping bands with long tail as found in our data sets (2). For the Raman data, an average spectrum over the three replicates was calculated and used for further analysis. Visual inspection of the baseline functions was conducted to identify any potential artifacts introduced by baseline subtraction. If necessary, the baseline parameters were adjusted to limit the introduction of any artifacts.

#### 2.4.1. Peak-fitting of spectra data

A plot was created of one case. After performing a visual inspection of the peaks, working spectral ranges were selected which included all the peaks of interest for this study and these were consistent between cows. The working spectra range included at least one of the peaks selected for the study. Lorentzian or pseudo-Voigt functions were used as the component functions in the fit. A component function was added for each selected peak in the working spectral range and was fitted for position, amplitude, and width manually to begin with. Due to the complexity of the spectral signal, components were only added for the most significant spectral features. In some cases, the unfitted components were accounted for by the tails of the added components which likely results in an over-estimation of the spectral width and amplitude (however, analysis of the peak parameter ratios limits any impact of these effects). Once a reasonable fit had been obtained manually, a non-linear least squares optimization using the scipy.optimize.curve_fit package (https://docs.scipy.org/doc/scipy/reference/generated/scipy.optimize.curve_fit.html) was used to find the optimal parameter values.

For each location, peak parameters (including peak location, amplitude, and width) were obtained, and in the case of Raman spectra averaged. The integrated intensities of characteristic bands were measured; integration was performed for each fitted component in the peak fitting function. The data were interpolated using a cubic spline for the Raman data (the FTIR data has a constant data interval). For the Raman data, we did not correct for spectrometer sensitivity or differential band-pass as these correction factors cancel when calculating band intensity ratios. The value used was the integrated area of the band as a direct proportion of the concentration of the specific chemical component. The integrated intensities of four characteristic bands measured by Raman spectroscopy were measured: ν_2_phosphate (422–454 cm^−1^), carbonate type B (1,046–1,110 cm^−1^), ν_1_phosphate (903–991 cm^−1^), and amide III (1,243–1,320 cm^−1^) (11, 13). Ratios of these bands areas resulted in the following parameters:

- Mineral/matrix ratio: ν_2_phosphate/amide III.
- Carbonate/phosphate ratio: carbonate/ν_1_phosphate.
- Crystallinity: 1/width of ν_1_phosphate.

Similarly, for FTIR the band assignments selected for analysis were: ν_1_ν_3_ phosphate (911–1,180 cm^−1^), ${\text{CO}}_{3}^{2-}$ (856–890 cm^−1^) and amide I (1,606–1,709 cm^−1^) (6, 11). Ratios of these band areas resulted in the following parameters:

- Mineral/matrix ratio: ν_1_ν_3_ phosphate/amide I.
- Carbonate/phosphate ratio: ${\text{CO}}_{3}^{2-}$/ν_1_ν_3_ phosphate.

Vibrational spectroscopy analysis is based on the principle that the integrated area of a band is directly proportional to the concentration of the specific molecular moiety giving rise to the specific band (6). Absolute measurements of Raman band intensities are difficult, especially in turbid media such as bone. Therefore, band intensity ratios are used when analyzing bone chemical composition (13). Bone matrix band assignments are mostly those of collagen type I (13).

### 2.5. Determination of the concentration of calcium and phosphorus

The absolute concentration of calcium and phosphorus was determined from 40 cows' bone samples (26 affected cases and 14 control cases). For each cow, a pooled sample (~300 mg) of the three bone locations (cortex, primary, and secondary spongiosa) was submitted to a commercial diagnostic laboratory (Gribbles Scientific, New Zealand) for determination of the percentage of bone calcium and phosphorus using inductively coupled plasma with mass spectrometry (NexION 2000B ICP Mass Spectrometer, PerkinElmer, USA). The affected and control cases used for this analysis were also used for Raman and FTIR spectroscopy.

### 2.6. Data statistical analysis

As the independent variables were not distributed normally, non-parametric tests of significance were used. An independent-samples Mann-Whitney U test was used to determine if there were any significant differences in the values of the ratios according to each bone location (cortex, primary, and secondary spongiosa) between affected and control cows. Results are presented as median, the *U* statistic, and *P* value. A *P* value of <0.05 was considered significant.

An independent-samples *t-*test was used to determine if there were any significant differences in humeral bone calcium and phosphorus percentage concentration between affected and control cows. Results are presented as mean ± standard deviation (SD), unless otherwise stated. All statistical analyses were done using SPSS statistics version 27 (IBM^®^ Corp., Chicago, IL, USA).

## 3. Results

### 3.1. Raman and FTIR spectra of bone samples

**Figure 2:**
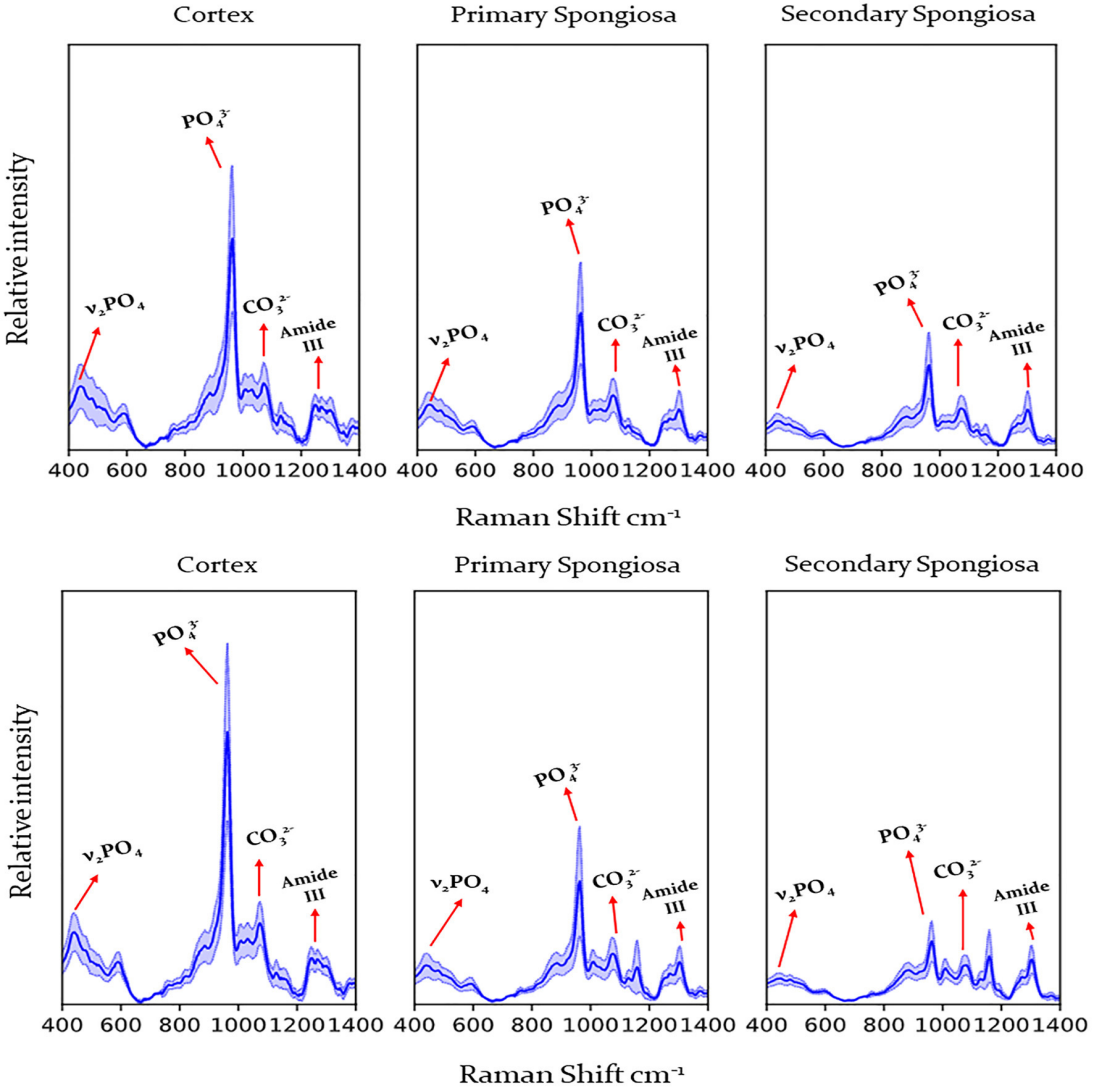
Raman spectra obtained from the humerus from cows with humeral fracture **(upper graph)** and control cows (no fractures) **(lower graph)** by location (cortex, primary, and secondary spongiosa). Labels indicate peaks used for calculation of mineral/matrix ratio, carbonate/phosphate ratio and crystallinity. Dark line indicates mean, pale blue line 95% confidence interval. ${\text{PO}}_{4}^{3-}$, phosphate; ${\text{CO}}_{3}^{2}$, carbonate.

**Figure 3:**
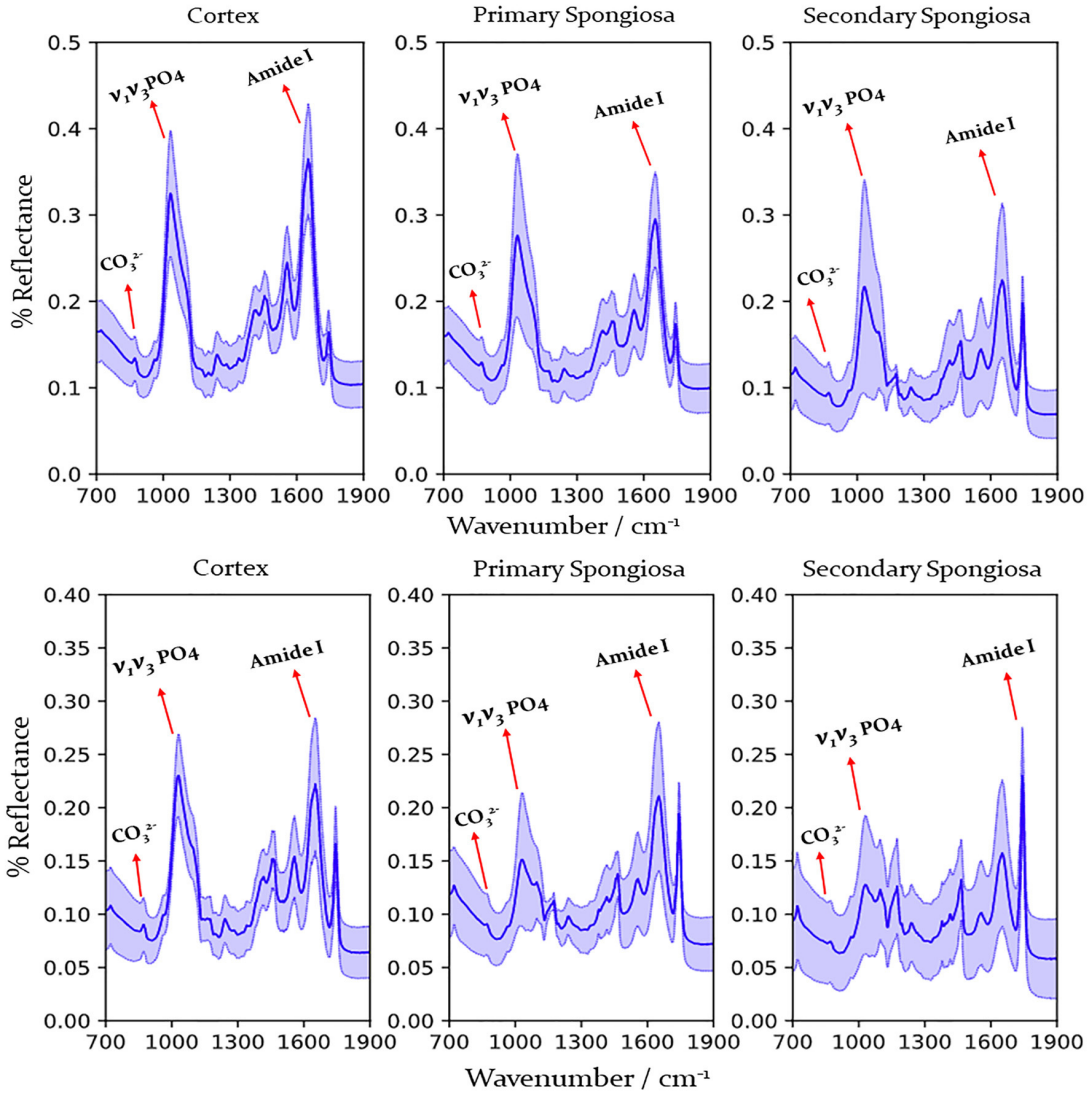
FTIR spectra obtained from the humerus from cows with humeral fracture **(upper graph)** and control cows (no fractures) **(lower graph)** by location (cortex, primary, and secondary spongiosa). Labels indicate peaks used for calculation of mineral/matrix and carbonate/phosphate ratio. Dark line indicates mean, pale blue line 95% confidence interval. ${\text{PO}}_{4}^{3-}$, phosphate; ${\text{CO}}_{3}^{2}$, carbonate.

Raman spectra were obtained from 67 affected cases and 14 control cases, and FTIR spectra were obtained from 66 affected cases (acquisition of spectra for one case was irregular hence that case was removed from this study) and 14 control cases. Figure 2 shows the representative spectrum of combined cases by fracture status and bone location for Raman spectroscopy and Figure 3 for FTIR spectroscopy. Results of the calculated ratios are represented in Tables 1, 2.

**Table 1:** Mann Whitney *U* test results of Raman spectra ratios and crystallinity by humeral bone location (cortex, primary, and secondary spongiosa) obtained from affected and control cows.

	**Case**	**Median**	**Mean rank**	***U***	***P* value**
**Cortex**					
Mineral/matrix	Affected (*n =* 67)	0.75	35.63	829.000	<0.005^*^
	Control (*n =* 14)	1.14	66.71
Carbonate/phosphate	Affected (*n =* 67)	0.12	34.07	933.000	<0.005^*^
	Control (*n =* 14)	0.21	74.14
Crystallinity	Affected (*n =* 67)	0.06	35.21	857.000	<0.005^*^
	Control (*n =* 14)	0.07	68.71
**Primary spongiosa**					
Mineral/matrix	Affected (*n =* 67)	0.99	39.81	549.000	0.318
	Control (*n =* 14)	1.08	46.71
Carbonate/phosphate	Affected (*n =* 67)	0.24	36.69	758.000	<0.005^*^
	Control (*n =* 14)	0.35	61.64
Crystallinity	Affected (*n =* 67)	0.06	34.36	9,140.000	<0.005^*^
	Control (*n =* 14)	0.07	72.79
**Secondary spongiosa**					
Mineral/matrix	Affected (*n =* 67)	0.67	46.70	84.000	<0.005^*^
	Control (*n =* 14)	0.34	13.71
Carbonate/phosphate	Affected (*n =* 67)	0.32	37.49	704.000	0.003^*^
	Control (*n =* 14)	0.51	57.79
Crystallinity	Affected (*n =* 67)	0.07	34.51	904.000	<0.005^*^
	Control (*n =* 14)	0.09	72.07

* *P* value < 0.05.

**Table 2:** Mann Whitney *U* test results of FTIR spectra ratios by humeral bone location (cortex, primary, and secondary spongiosa) obtained from affected and control cows.

**Calculated ratio**	**Case**	**Median**	**Mean Rank**	**U**	***P* value**
**Cortex**					
Mineral/matrix	Affected (*n =* 66)	0.96	37.70	277.000	0.019^*^
	Control (*n =* 14)	1.23	53.71
Carbonate/phosphate	Affected (*n =* 66)	0.12	43.33	275.000	0.018^*^
	Control (*n =* 14)	0.09	27.14
**Primary spongiosa**					
Mineral/matrix primary	Affected (*n =* 66)	0.88	44.36	207.000	0.001^*^
	Control (*n =* 14)	0.58	22.29
Carbonate/phosphate	Affected (*n =* 66)	0.25	36.15	175.000	<0.005^*^
	Control (*n =* 14)	0.53	61.00
**Secondary spongiosa**					
Mineral/matrix	Affected (*n =* 66)	0.84	44.30	211.000	0.001^*^
	Control (*n =* 14)	0.46	22.57
Carbonate/phosphate	Affected (*n =* 66)	0.09	33.50	0.000	<0.005^*^
	Control (*n =* 14)	0.49	73.50

* *P* value < 0.05.

#### 3.1.1. Bone cortex

Bones from affected cases showed a significantly lower mineral/matrix ratio as measured by Raman and FTIR spectroscopy (*P* < 0.005 and *P* = 0.019 respectively). The carbonate/phosphate ratio was significantly lower in the cortex of affected cases compared to control cases when measured by Raman spectroscopy (*P* < 0.005). In contrast, FTIR results showed a lower carbonate/phosphate ratio in control cases compared to affected cases (*P* = 0.018). Finally, crystallinity determined using Raman spectroscopy was significantly higher in control cases compared to affected cases (*P* < 0.005).

#### 3.1.2. Bone primary spongiosa

Bones from affected cases had significantly lower carbonate/phosphate ratio when measured by both Raman and FTIR spectra analysis (*P* < 0.005 and *P* < 0.005 respectively). The use of Raman spectra showed no evidence for a difference in the mineral/matrix ratio (*P* = 0.318), contrary to when FTIR spectra was used, which showed a lower mineral/matrix ratio in control cases (*P* = 0.001). Finally, crystallinity was determined using Raman spectra and was significantly higher in control cases compared with affected cases (*P* < 0.005).

#### 3.1.3. Bone secondary spongiosa

Bone from affected cases had a significantly lower carbonate/phosphate ratio when measured by Raman and FTIR spectra analysis (*P* = 0.003 and *P* ≤ 0.005 respectively). The mineral/matrix ratio was significantly lower in bones from control cases when measured by both Raman (*P* < 0.005) and FTIR (*P* = 0.001) spectroscopy. Crystallinity was significantly higher in control cases compared with affected cases (*P* < 0.005).

### 3.2. Calcium and phosphorus concentration

Table 3 shows the mean ± SD percentage concentration of bone calcium and phosphorus content and the results of the statistical analysis. There were no significant differences in the percentage of calcium and phosphorus concentration in bone from cows with humeral fracture compared with control humeri (*P* = 0.5 and *P* = 0.6 respectively).

**Table 3:** Mean ± SD of the calcium and phosphorus percentage concentration in humeral samples from affected and control cows.

	**Case**	**Mean ±Std. deviation**	***P* value**
Bone Calcium (%)	Affected (*n =* 26)	19.38 ± 2.41	0.5
	Control (*n =* 14)	18.91 ± 1.56
Bone phosphorus (%)	Affected (*n =* 26)	8.96 ± 1.10	0.6
	Control (*n =* 14)	8.81 ± 0.78

## 4. Discussion

Results of this study demonstrate that there are significant differences in the relative chemical composition (bone quality changes) of cortical and trabecular bone from affected and control cows when measured by Raman and FTIR spectroscopy. More importantly, significant change in the bone tissue mineral component quality of the cortical bone is substantially reduced, thus contributing to reduced bone strength.

### 4.1. Mineral/matrix ratio

The mineral/matrix ratio has been used extensively to study bone quality and strength and is an alternative method for reporting bone density (6, 9, 14, 15). This measure accounts for differences in the amount of bone organic matrix and so is of great value for assessing bone quality and is known to be reduced in humans with osteoporosis (6, 10). Compared to controls, cases with humeral fractures had a significantly lower mineral/matrix ratio in cortical bone when measured by both Raman and FTIR spectrometry. These findings demonstrate altered and decreased mineralization in bones of cows with humeral fractures, further elucidating components of their osteoporosis and offering further insight mechanisms of the catastrophic humeral fractures.

Furthermore, the mineral/matrix ratio can provide information on the mechanisms of bone loss that lead to osteoporosis (16). A lower mineral/matrix ratio is reported in iliac crest bone biopsies from women and men with high-turnover osteoporosis (defined as an increased number of resorption surfaces and higher than the normal number of osteoclasts) compared to bone biopsies samples from women and men with low-turnover osteoporosis and normal bone activity (16). These results corroborate the findings from a previous analysis done on this same set of bone samples, which found that bones from cows with humeral fractures had increased abnormal resorption of cortical bone and increased bone resorption in this distal humerus (4). Furthermore, peripheral quantitative computed tomography data has found a reduction in cortical bone mineral density in the mid-diaphysis of the humerus of fractured cows (3). Less mineralized bone has less resistance to fracture (9).

Moreover, a strong interdependence is described between reduced mineral/matrix ratio, bone bending stiffness (a measure of the bone resistance to deformation under an applied load), and failure moment in cortical bone of rats with reduced bone mineral density (14, 17). The findings of the current study indicate that in affected cows cortical bone strength and quality are significantly compromised with less organic matrix and lower mineralization due to active bone remodeling, which likely results in decreased bending stiffness and decreased resistance to fracture.

A decreased mineral/matrix ratio was found in the secondary spongiosa (when measured by Raman and FTIR) and primary spongiosa (measured by FTIR) of control cows compared with affected cows. These results suggest that in control cows the primary spongiosa and secondary spongiosa are the sites of active bone remodeling activity. This is consistent with the knowledge that, although resorption occurs normally in both cortical and trabecular bone, the rate of remodeling is much greater in trabecular bone (20%) compared to cortical bone (5%) (6, 18). It appears that in affected cows, trabecular bone is not readily available for remodeling and for Ca supply, meaning animals are relying on the cortical bone for these processes. The histological findings that cows with humeral fractures have significantly reduced trabecular bone density and abnormal trabecular bone architecture which may not be readily available for re-modeling, support this hypothesis (4).

There is an important effect of tissue age on the mineral/matrix ratio with the ratio increasing with tissue age when measured on the bone trabecular surface of healthy females (6, 15). Using this ratio to determine tissue age, older tissue was found in the cortex and younger bone in the secondary spongiosa of control cows. In contrast, this relationship was reversed in affected cows with the oldest tissue being found in metaphyseal bone followed by the cortex. This is another finding that supports the switch in remodeling activity from the trabecular bone to cortical bone in affected cows.

It is important to remember that bone tissue is a very heterogeneous material, different bones in the same individual/animal and different locations within the same bone may be undergoing active bone remodeling or be completely inactive (6, 14). Because tissue homogenates were used in this study variability in tissue microstructure (temporal and spatial organization) was not considered when interpreting and comparing values. Lastly, although both spectroscopic techniques are complementary to each other, they use different techniques for obtaining bands and intensities which could lead to some of the variability in the ratios reported in this study (13).

### 4.2. Carbonate/phosphate ratio

The carbonate/phosphate ratio measures the amount of carbonate substitution in bone (7). Pure apatite crystals found in nature consist of calcium, phosphate, and hydroxyl ions (6). In contrast, apatite crystals found in bone are highly substituted, are poorly crystalline, and vary according to the location in bone (cortical vs. trabecular) and the bone metabolic state (6, 19). Carbonate can substitute for either hydroxyl ions (type A substitution) or phosphate (type B substitution) (6, 11). Type B substitution was measured in this study.

The carbonate/phosphate ratio impacts bone mechanical properties and in very old subjects can be associated with deterioration in the structural and mechanical material properties of bone (7, 10, 20). For example, carbonate substitution (carbonate/phosphate ratio) was significantly higher in skeletally mature, old murine bones compared to skeletally mature, young murine bones, and the carbonate/phosphate ratio positively correlated with bone plasticity (deformation) index (20). McCreadie et al. (9) found a greater carbonate/phosphate ratio in the iliac crest cortical bone from women (>50 years old) with osteoporosis when measured by Raman spectrometry and concluded that the carbonate/phosphate ratio was an important determinant in the occurrence of osteoporotic fracture (9).

In the present study, significant differences were found in all three locations examined (cortex, primary, and secondary spongiosa) and with both spectroscopic methods used. Higher carbonate substitution was found in the primary and secondary spongiosa of control cows compared with affected cows. This increased carbonate substitution may contribute to bone mechanical strength in control cows.

As such, less carbonate substitution in affected cows may be an important contributory factor to spontaneous humeral fractures. Is should be noted that in the human study this ratio was performed on iliac crest bone and not on a long bone and that murine skeletal remodeling may be physiologically different to cows.

Changes in the chemical composition of bones are irregular (non-linear), and complex, and there is a wide range of other mechanisms that can influence tissue chemical composition that need to be considered (9). These include genetically/environmentally determined differences in the chemistry of bones and bone damage (9). Damage itself may result in changes from location to location within the same bone thus influencing bone remodeling and repair which may greatly influence chemical composition within the same bone (9). Several of these mechanisms may have confounded the results of this study. For example, these spectra were measured from dairy cows that have recently calved and have physiological mechanisms dedicated to milk production that could have affected the chemical composition of these bones. Since control cases were the same age and lactating it is believed we controlled for any effect of age or lactation. However, milk production, milking frequency, and body condition score, all of which can potentially influence the chemical composition of bone, were variables that we were unable to control for and could have resulted in some bias in the results.

### 4.3. Crystallinity

The term crystallinity refers to the size and shape of bone apatite crystals (6, 21). Carbonate substitutions and crystallinity are closely correlated, whereby the type and extent of substitutions influence crystal solubility, size, and shape (6). Crystal size also changes with the stage of mineralization (less mature mineral, less crystallite size), with crystallinity increasing with age, suggesting a more ordered crystal lattice (7, 11).

Results from the current study showed crystallinity was significantly reduced in the cortex, the primary and secondary spongiosa, of bones from affected cows compared to control cows. In cortical bone increasing crystallinity is positively correlated with tissue-level strength and stiffness and negatively correlated with ductility (the ability of a material to be plastically deformed without fracture) (7, 21).

Therefore, reduced crystallinity in bone tissue from affected cows may contribute to decreased bone strength in all locations (cortex, primary, and secondary spongiosa) due to mineral crystals that are of reduced size, less mature, less mineralized, and less ordered, likely resulting in the weak bone in affected cows, predisposing them to humeral fractures.

### 4.4. Bone calcium and phosphorus content

The phosphorus and calcium percentages were similar in bone from affected and control cows, with no significant differences between groups. In animals with osteoporosis, there is a reduction in the quantity of bone, which is proportional to a reduction in the mineral composition of bone, as such changes in proportion are not likely to be present (22). While this finding differs from the decreased mineral/matrix ratio in cortical bone of affected cows, we used whole bone samples to measure calcium and phosphorus content as opposed to for example bone ash. Holst et al. (23) used rib bone to assess bone mineralization in range cattle and found that ash density (mg/mL), not phosphorus or calcium percentage, was better for identifying cattle with impaired bone mineralization. Additionally, while the mineral/matrix ratio was lower in cortical bone of affected cows. It was greater in metaphyseal bone of affected cows, thus by using whole bone (cortical and metaphyseal) we fail to detect these differences.

## 5. Conclusion

Raman and ATR-FTIR spectra analysis of bone from primiparous cows with humeral fractures compared to age-matched control cows indicates that in cows with humeral fractures there is a significant difference in the relative bone chemical composition and quality. Affected bone showed a significantly reduced mineral/matrix ratio, increased bone remodeling, newer bone tissue with lower mineralization and, lower carbonate substitution, and reduced crystallinity.

All these changes in the bone chemical composition of affected cows (especially in cortical bone) significantly impact bone quality, reduce bone strength, and likely contribute to the increased incidence of spontaneous fractures in first calving dairy cows in New Zealand.

## Data availability statement

The original contributions presented in the study are included in the article/supplementary material, further inquiries can be directed to the corresponding author.

## Ethics statement

Ethical review and approval was not required for the animal study because samples were collected by the farmers veterinarian as part of a diagnostic investigation into the cause of the fracture and sent to Massey University as such animal ethical approval was not required. Written informed consent for participation was not obtained from the owners because owners submitted samples and filled in a submission form as part of a diagnosis investigation, as such written consent was not required.

## Author contributions

AW-M, KD, and MW were responsible for the conception of the study. AW-M was responsible for data collection, data interpretation, and manuscript drafting and editing. AW-M and MW were responsible for data analysis. RN, KL, PB, CR, MW, and KD were responsible for manuscript revisions. All authors contributed to the article and approved the submitted version.
